# Non-suicidal self-injury in Chinese adolescents: longitudinal associations with negative life events and life satisfaction

**DOI:** 10.3389/fpsyt.2024.1470371

**Published:** 2025-03-28

**Authors:** Zhansheng Xu, Yaxin Kong, Yang Yang, Mingyangjia Tian, Yu Liu, Lin Lin

**Affiliations:** ^1^ Key Research Base of Humanities and Social Sciences of the Ministry of Education, Academy of Psychology and Behavior, Tianjin Normal University, Tianjin, China; ^2^ Faculty of Psychology, Tianjin Normal University, Tianjin, China; ^3^ Center of Collaborative Innovation for Assessment and Promotion of Mental Health, Tianjin, China; ^4^ Collaborative Innovation Center of Assessment Toward Basic Education Quality, Beijing Normal University, Beijing, China; ^5^ Intelligent Laboratory of Child and Adolescent Mental Health and Crisis Intervention of Zhejiang Province, Zhejiang Normal University, Jinhua, Zhejiang, China; ^6^ Department of Psychology, Zhejiang Normal University, Jinhua, Zhejiang, China

**Keywords:** adolescence, negative life events, life satisfaction, non-suicidal self-injury, longitudinal study

## Abstract

**Objectives:**

The relationship between negative life events, life satisfaction, and nonsuicidal self-injury has been demonstrated in adolescence, but no study has examined the longitudinal associations between the three over time. The present study hypothesized that negative life events play a mediating role in the relationship between life satisfaction and non-suicidal self-injury over time.

**Methods:**

A total of 268 junior high school students participated in three questionnaires with an interval of half a year and completed questionnaires investigating the Adolescents Self-Harm Scale, the Adolescent Self-Rating Life Events Checklist, and the Satisfaction with Life Scale.

**Results:**

The results indicated a significant predictive role of negative life events in non-suicidal self-injury over time and the prospective effect of life satisfaction on negative life events in adolescence. Negative life events play an intertemporal mediation in the relationship between life satisfaction and non-suicidal self-injury.

**Conclusion:**

As a clear non-suicidal self-injury risk factor, negative life events can significantly predict non-suicidal self-injury in adolescence whether it is horizontal or vertical. Junior high school students who are exposed to more negative life events are more likely to have non-suicidal self-injury behaviors and the negative life events have a certain lagging effect on non-suicidal self-injury. Due to the prospective effect of life satisfaction on negative life events in adolescents, negative life events play a mediation in the relationship between life satisfaction and non-suicidal self-injury over time.

## 1 Introduction

Non-suicidal self-injury (NSSI) is defined as direct, intentional damage to one’s own body tissues with no suicidal intent, including cutting or carving the skin and so on (1). It’s an important risk factor for suicide attempts and other psychological problems (1) and is considered to be an important public health problem among adolescents (2). In adolescent samples worldwide, the prevalence of NSSI is about 19% (range 3–52%), which is much higher than adults (about 13%) (3, 4). A study pooled adolescents aged 10 to 19 years (21.4% of female adolescents and 13.7% of male adolescents) from 17 different countries in North America, Australia, Europe, and Asia with a prevalence of NSSI of 17.7% (5). In China, the lifetime prevalence of Non-Suicidal Self-Injury (NSSI) among children and adolescents remains high, estimated to be 29.3% (28.5-30.1) among primary school students, 25.3% (20.7-41.9) among junior high school students, and 32.8% (26.6-35.6) among high school students (6). Therefore, exploring the influencing factors of adolescent NSSI is of great significance for preventing adolescent NSSI and even for subsequent possible psychological problems.

In recent years, with the development of various theories on NSSI, researchers have found that there are many factors related to NSSI. Stressful life events are the risk factor of self-injury that is often discussed in current studies and life satisfaction also plays a role in the formation mechanism of self-injury (7, 8). Therefore, we designed this study to investigate the relationship and orientation between negative life events, life satisfaction, and NSSI by using a one-and-a-half year longitudinal design with junior high school students as samples.

### 1.1 Negative life events and NSSI

Negative life events refer to stressful life events that may bring negative physiological and psychological reactions to adolescents (9). Previous studies have shown that negative/stressful life events could lead to individuals’ psychopathological risks, such as depression, impulsive aggression, suicidal ideation, and suicide attempts and so on (10, 11). In a meta-analysis (12), the psychopathological stress exposure model (13) has been applied to non-suicidal self-injury and has been validated by relevant studies. For example, some studies have found that a higher incidence of stressful life events is associated with higher NSSI behaviors (8, 14–16). The integrated theoretical model (17) also emphasized that stressful life events are risk factors for self-injury, especially those with difficulty in emotional regulation may adopt self-injury as a stress relief method after experiencing negative life events.

Although these theories all emphasized the importance of stressful life events in the occurrence of NSSI, there is still a problem in recent studies that the causal relationship of NSSI with stressful life events is not yet clear. Although some longitudinal studies have found that negative life events can affect NSSI among adolescents over time (18–20), some studies have found that adolescents with NSSI experience more negative life events (21), and that the NSSI of late adolescent girls lead to more interpersonal stress events over time (22). Therefore, to explore the relationship between negative life events and NSSI is essential to subdivide the existing causal relationships and improve the current theoretical model.

### 1.2 Life satisfaction and NSSI

Life satisfaction can be defined as an individual’s overall evaluation of his or her life (23) and is one of the most important aspects of subjective well-being (24). It is regarded as an aspect of individuals’ mental health (25) and associated with many mental health problems, such as depressive symptoms, suicide and so on (26–30). Some studies also found that dissatisfaction with oneself, interpersonal relationship and the environment is significantly associated with NSSI (7, 31) and life dissatisfaction is a risk factor for NSSI (32, 33). However, there are still some differences in the study of the relationship between life satisfaction and NSSI. For example, Self-injured people’s life satisfaction decreased, but in the follow-up logistic regression analysis, life satisfaction can’t distinguish self-injured people (34). This suggests that the effect of life satisfaction on self-injure is more complex and may interact with other factors. In addition, whether life satisfaction changes after NSSI, especially for adolescents, has not been studied yet.

### 1.3 Negative life events and life satisfaction

Individuals’ judgment of life satisfaction is based on the objective conditions of life and the life events they experience (35, 36). A Meta-Analysis showed that life events have strong effects on life satisfaction (37). There was a significant negative correlation between major negative life events and life satisfaction, and it could predict life satisfaction well (38, 39). Some studies found life satisfaction is not only an individual’s subjective perception of life but also a predictor of an individual’s future development and even experience. For example, longitudinal studies have also found that life satisfaction predicts future health outcomes and survival rates (40, 41). Luhmann et al. (42) found that life satisfaction is an important predictive factor for negative life events such as divorce and unemployment through a five-year follow-up study.Individuals with low life satisfaction change their life circumstances through temporary mechanisms (e.g., by ending a current and negative relationship). Also, life satisfaction affects life events through stable mechanisms, such as personality. Wootton et al. (43) found genes associated with positive qualities such as subjective well-being may allow individuals to look for an environment where positive life events occur and avoid negative life events. However, there is no study on the relationship between negative life events and life satisfaction among adolescents’ NSSI.

### 1.4 Hypotheses

In order to solve these gaps and problems in the existing literature, this study used a one-and-a-half-year longitudinal design of junior high school students as samples to test the relationship and direction between negative life events, life satisfaction, and NSSI. Regarding the relationship between life events and NSSI, we hypothesized that life events can predict NSSI positively over time; Regarding the relationship between life satisfaction and negative life events, we assumed that they can predict each other over time; Regarding the relationship between life satisfaction and NSSI, we assumed that negative life events play a mediating role in the relationship between life satisfaction and NSSI over time.

## 2 Methods

### 2.1 Participants

The current longitudinal study consisted of three half-yearly measurement points with six months intervals. At Time 1 (T1), the sample consisted of 268 students in grade one of junior high school (48.5% female) between the ages of 10 and 16 (*M* = 12.69; *SD* = 0.71). At Time 2 (T2), 227 adolescents (*M* = 13.13; *SD* = 0.66; 51.5% female) and at Time 3 (T3), 211 students (*M* = 13.62; *SD* = 0.65; 50.7% female) participated. A total of 206 students participated in all three measurement points. Data were collected in a secondary school in Tianjin, China. All participants were Chinese. Percentages of missing responses across the variables at each time-point are provided in **Table 1**.

**Table 1:** Percentages of missing responses across the variables at each time-point.

		T1	T2	T3
Self-injury	Sample size	268	227	211
	Missing responses (%)	0 (0%)	41 (15.3%)	57 (21.3%)
Negative life events	Sample size	268	228	211
	Missing responses (%)	0 (0%)	40 (14.9%)	57 (21.3%)
Life satisfaction	Sample size	268	229	212
	Missing responses (%)	0 (0%)	39 (14.6%)	56 (20.9%)

Generally, the sample size should be more than 5 times the number of questions in the questionnaire (44). Our study questionnaire had 38 questions, so the sample size should be greater than 175.The sample sizes of all three of our questionnaire collections met this requirement.

### 2.2 Procedure

With the consent of the school and parents, we conducted a questionnaire survey among the junior high school students in the first grade of the whole junior high school. The study took place during school hours, with the researchers present at all times. Researchers are all in-school graduate students and have received unified training. Each student received an informed consent form, the questionnaire booklet and a set of world-famous school bookmarks as compensation. A unique code was assigned to each student and was used throughout the entire study to ensure anonymity. The study was approved by the Ethics Committee of Tianjin Normal University.

### 2.3 Measures

#### 2.3.1 Non-suicidal self-injury

At each time-point, NSSI was assessed using three questions: 1. In the past six months, have you ever intentionally cut, burned, cut yourself, or hurt yourself in any other way? (yes/no); 2. In the past six months, have you ever deliberately pricked a wound to prevent it from healing? (yes/no);3. In the past six months, have you ever intentionally let someone beat or bite you in order to hurt your body? (yes/no). If the subjects choose “yes” score 1, “no” score 0, and the total score of the last three questions as the criteria for judging the occurrence of non-suicidal self-injury in the past six months.

#### 2.3.2 Negative life events

The Adolescent Self-Rating Life Events Checklist (ASLEC) was used to assess individuals’ subjective suffering from stressful life events experienced during the past 6 months. The ASLEC is a 27-item self-report questionnaire consisting of 5 types of stressful life events: interpersonal problems, school-related problems, parental problems, punishment and loss, and health and adaptation problems (9). Questions are answered on a 6-point scale (0 = did not occur to 5 = occurred and was extremely stressful). Higher total scores on the scale represent a greater number of stressful life events experienced in the past year. The reliability and validity of the ASLEC in China have been well established in hundreds of studies. Internal consistency (Cronbach’s alpha) for the ASLEC at three time-points was.93,.95,.05.

#### 2.3.3 Life satisfaction

The Satisfaction with Life Scale (SWLS) was used to assess life satisfaction. The SWLS (23) is a 5-item self-report measure of overall satisfaction with life. Questions are answered on a 7-point Likert scale (1 = strongly disagree to 7 = strongly agree). Responses are summed to provide an overall score. The reliability and validity of the SWLS have been well established in hundreds of studies. The reliability and validity of SWLS among Chinese are outstanding (45). Internal consistency (Cronbach’s alpha) for the SWLS at three time-points was.75,.77,.84.

### 2.4 Data analysis

We used multiple imputations to handle missing data at follow-up (46). We created 100 multiply imputed datasets (47) using the Markov Chain Monte Carlo sequence (convergence value was set to 0.001) and unrestricted variance-covariance technique. This state of the art missing data handling increases the power of our analyses and avoids biased estimates that would be obtained with conventional missing data handling strategies (46).

**Figure 1:**
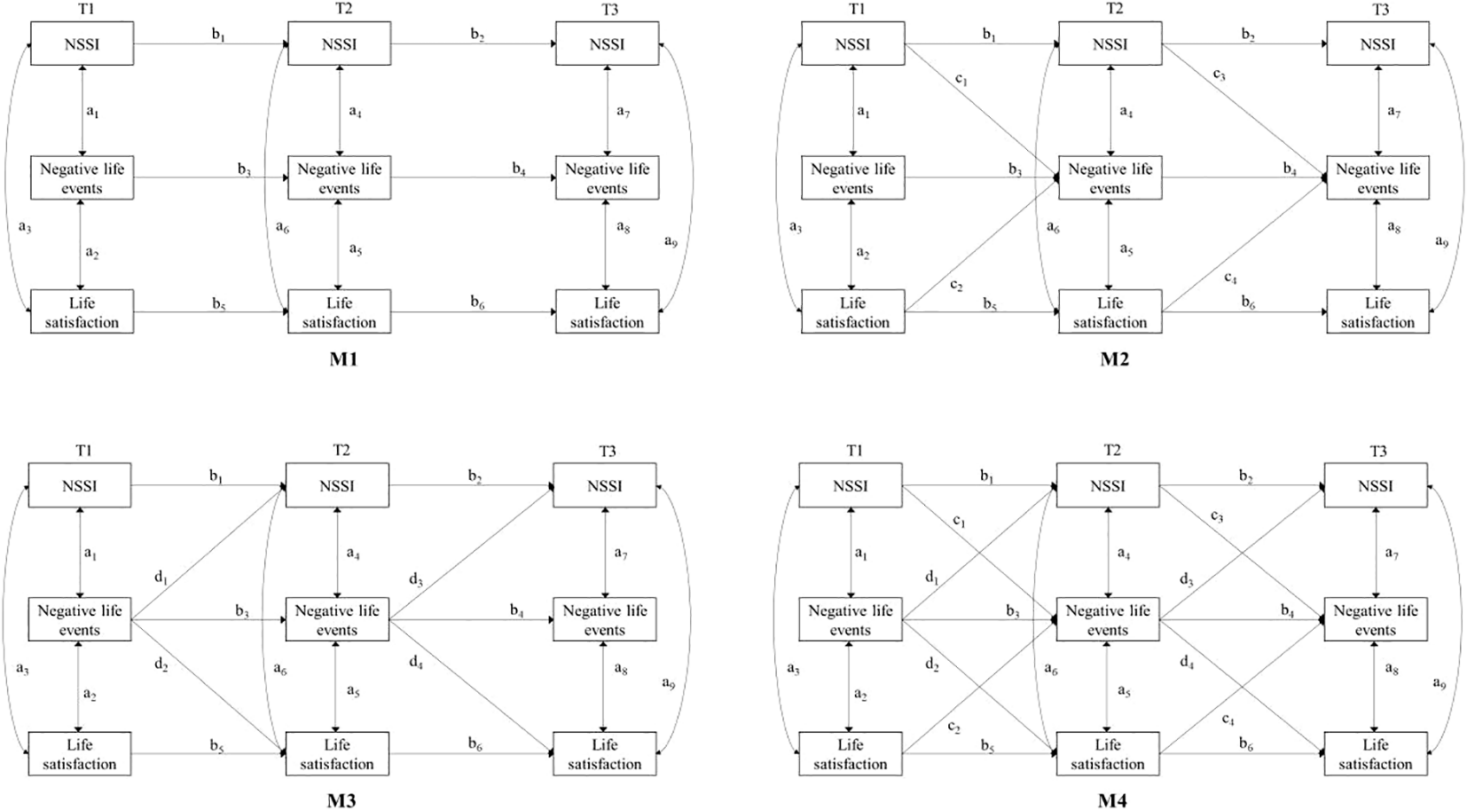
Cross-lagged model tested in the present study with initial associations (a1 - a3), correlated relative change (a4 - a9), six-months relative stability paths (b1 - b6), and cross-lagged paths in the first time interval (c1, c2; d1, d2) and the second time interval (c3, c4; d3, d4).

Multiply imputed datasets were generated using Mplus 7.4 (48), resulting in a final data set with 268 participants at each wave (which equals the number of participants at T1). The robust maximum likelihood estimator (MLR) method was used for model estimation. According to the analysis method of longitudinal data, four models were tested in turn: M1 is a baseline model that only includes autoregression which tested initial associations, correlated relative change, and six-months relative stability among students’ NSSI, negative life events, and life satisfaction at T1, T2, and T3; M2 added the influence path of NSSI and life satisfaction at the previous time point to the negative life events at the next time point on the basis of M1; On the basis of M1, M3 added paths similar to the cross-lag paths in M2 but in the opposite direction, that is, the negative life events at the previous time point to NSSI and life satisfaction at the next time point; M4 is a full model that includes all the paths in the above model. The models tested are shown in **Figure 1**. Five criteria were used to fit the evaluation model: χ^2^, CFI, TLI, RMSEA and SRMR. According to (49), CFI and TLI should be greater than.90 to ensure the fitness of the model, RMSEA and SRMR should be less than.08 to indicate an acceptable fit.

## 3 Results

### 3.1 Missing data analysis

An analysis was made of the missing subjects and those who continued to participate in the study. Results showed there were non-significant difference in NSSI (*t_(266)_* = 0.75), negative life events (*t_(266)_* = 0.99) at T1 and gender (*χ* ^2^ = 2.66, *df* = 1). There was a significant difference in age (*t_(266)_* = 3.56, *p* < 0.001), the missing subjects (*M* =12.97, *SD* = 0.80) were significantly older than those who continued to participate in the study (*M* =12.61, *SD* = 0.66). A marginal significant difference in life satisfaction (*t_(266)_* = -1.97, *p* = 0.04994) at T1 was found, the life satisfaction of missing subjects (*M* =20.20, *SD* = 5.88) were significantly lower than those who continued to participate in the study (*M* =22.00, *SD* = 6.39).

### 3.2 Common method bias test

Harman single factor test was used to test the common method bias of variables at each time point (50). For T1, exploratory factor analysis showed that there were 7 factors with characteristic roots greater than 1, and the interpretation rate of the first factor was 9.66% (< 40%); For T2, there were 8 factors with characteristic roots greater than 1, and the interpretation rate of the first factor was 11.57% (< 40%); For T3, there were 5 factors with characteristic roots greater than 1, and the interpretation rate of the first factor was 12.31% (< 40%); This results indicated that there was no obvious common method deviation in all data of this study.

### 3.3 Descriptive analysis

First, prevalence of NSSI at T1 was around 20.5% and 6 month-prevalence at T2 and T3 was 17.6% and 19.0%, respectively. Second, there was no age difference in all variables measured at three time points. Third, gender differences in NSSI at T1 (*t_(266)_* = -4.23, *p* <.001) and T2 (*t_(225)_* = -2.21, *p* <.05) were significant. Girls experienced more NSSI than boys at T1 (*M_F-T1_* = .50, *SD* = .85; *M_M-T1_* = .14, *SD* = .49) and T2 (*M_F-T2_* = .37, *SD* = .75; *M_M-T2_* = .17, *SD* = .56). **Table 2** displayed descriptive statistics and correlation coefficients among all study variables.

**Table 2:** Descriptive statistics of NSSI (range 0 – 3), NLE (range 0 – 130) and LS (range 5–35) and correlations among variables.

	1	2	3	4	5	6	7	8	9
1. NSSI T1	1								
2. NSSI T2	0.61^***^	1							
3. NSSI T3	0.48^***^	0.63^***^	1						
4. NLE T1	0.22^***^	0.30^***^	0.30^***^	1					
5. NLE T2	0.21^**^	0.30^***^	0.29^***^	0.61^***^	1				
6. NLE T3	0.13	0.16^*^	0.28^***^	0.57^***^	0.73^***^	1			
7. LS T1	-0.14^*^	-0.22^**^	-0.05	-0.21^***^	-0.28^***^	-0.23^**^	1		
8. LS T2	-0.22^**^	-0.25^***^	-0.25^***^	-0.23^***^	-0.31^***^	-0.30^***^	0.61^***^	1	
9. LS T3	-0.11	-0.21^**^	-0.17^*^	-0.15^*^	-0.19^**^	-0.20^**^	0.41^***^	0.59^***^	1
*M*	0.32	0.27	0.30	40.17	32.23	36.87	21.59	22.13	21.95
*SD*	0.71	0.67	0.70	26.23	26.30	27.86	6.31	6.02	6.37

NSSI, Non-suicidal self-injury; NLE, Negative life events; LS, Life satisfaction.

^*^ *p*<0.05; ^**^ *p*<0.01; ^***^ *p*<0.001.

### 3.4 Cross-lagged analysis

The final fit index of each model was shown in **Table 3**. The M4 model had a good fit (χ^2^ = 24.55, df = 13, RMSEA = 0.06, SRMR = 0.04, CFI = 0.98; TLI = 0.94) and performed best among the four models.

**Table 3:** Model fit index of four models.

	*χ^2^*	*df*	RMSEA	SRMR	CFI	TLI
M1	50.55	21	0.07	0.09	0.94	0.91
M2	42.71	17	0.08	0.08	0.95	0.90
M3	31.71	17	0.06	0.05	0.97	0.94
M4	24.55	13	0.06	0.04	0.98	0.94

**Figure 2:**
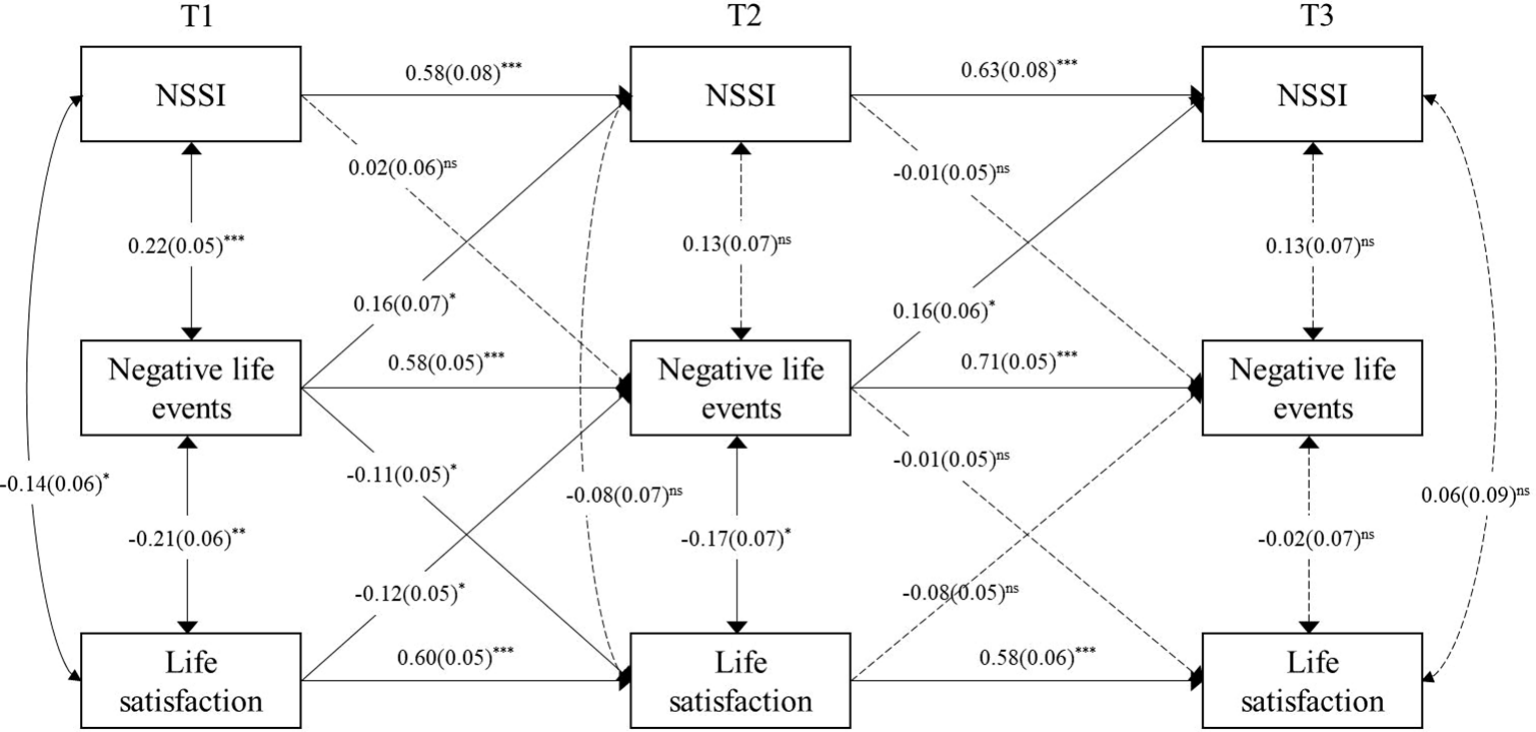
Cross-lagged path model with gender as a covariate with standardized path coefficients and standard errors. ^ns^ *p*>0.05, ^*^ *p*<0.05; ^**^ *p*<0.01; ^***^ *p*<0.001.

The M4 model better reflected the relationship between variables, so it is ultimately retained as the best model. **Figure 2** presented the standardized estimates averaged over 100 imputed datasets in M4. At T1, all within-time associations were significant. At T2 and T3, all relative correlated change paths were non-significant, with the exception of the significant negative relation between negative life events and life satisfaction at T2. Results showed a unidirectional association at both intervals between negative life events and NSSI: negative life events at T1/T2 positively predicted NSSI at T2/T3. In addition, a two-way association was found only in the first interval between life satisfaction and negative life events: life satisfaction at T1 negatively predicted negative life events at T2; negative life events at T1 negatively predicted life satisfaction at T2. Finally, A cross-time mediation effect was found: life satisfaction at T1 negatively predicted negative life events at T2 and then influence NSSI at T3.

## 4 Discussion

The results of our study indicated a significant predictive role of negative life events in the development of NSSI over time. Furthermore, we found that life satisfaction in adolescence has a prospective effect on the occurrence of negative life events. Importantly, negative life events emerged as an intertemporal mediator in the relationship between life satisfaction and NSSI, suggesting that they play a crucial role in linking these two constructs.

### 4.1 Gender difference in adolescent NSSI

Gender differences in adolescents’ NSSI appear in the first two surveys of this study, girls experienced more NSSI than boys. This is consistent with previous findings that NSSI in adolescence and early adulthood more commonly affects young females (51, 52). There are many differences between adolescent boys and girls in physical and psychological development, especially girls are more likely to adopt NSSI behavior in early adolescence because of their sensory experience of more negative information, like daily peer hassles and so on (53, 54). With the development of their cognitive level and psychological ability, girls will gradually learn to better solve the problem and the experience of positive emotions may be higher than boys (55). It may be an important reason for the disappearance of gender differences in NSSI behavior in the third data collection of this study.

### 4.2 Negative life events and adolescent NSSI

In line with part of expectations, cross-lagged analyses indicated a unidirectional positive association between negative life events and NSSI over time. The unidirectional relationship suggests that NSSI in adolescents may be the consequence of experiencing negative life events. These findings are consistent with the results of previous longitudinal studies on the relationship between negative life events and self-injury (19, 20). On the other hand, they also confirm the prospective risk factors of self-injury in adolescents with negative life events. Experiential Avoidance Model (56) believes that the main function of self-injury lies in avoiding or escaping from an individual’s unwanted internal experience or behavior. Following a negative event that evokes aversion and other emotions, individuals may engage in self-harmful behaviors due to various influences, seeking to escape or lessen the unpleasant feelings. Although self-injury makes individuals escape the negative emotions brought by negative events, this kind of behavior may play a negative reinforcement role, strengthen the relationship between negative event stimulation and self-injury, and make individuals continue to adopt self-injury behavior as an automated avoidance response when facing negative life events or unpleasant emotional experiences again. This research finding is consistent with the viewpoint of the Integrated Theoretical Model of self-injury (17). The model emphasizes that stressful life events are significant risk factors for self-injurious behaviors. However, no effect of self-injury on negative life events was found in this study, which was contrary to previous studies. Negative life events in this study are a broad concept, but Burke et al. (22) mainly discussed the relationship between interpersonal relationships and self-injury.

### 4.3 The effect of negative life events between life satisfaction and adolescent NSSI

Cross-lagged analyses indicated a two-way association in the first interval between life satisfaction and negative life events. Specifically, experiencing negative life events at T1 is associated with lower life satisfaction at T2. The bottom-up (situation) model of life satisfaction believes that people’s life satisfaction is mainly affected by the situations, events and circumstances they are in. For example, job satisfaction, marital satisfaction, health status, and social relationships are factors that affect people’s life satisfaction (57). Negative life events belong to situational factors and are an important part of the bottom-up model. A study investigated 146 young people using a 14-day daily diary design and found that daily negative life events had a negative predictive effect on daily life satisfaction (58). Conversely, higher life satisfaction at T1 is associated with fewer negative life events at T2. On the one hand, this result validates the prospective effect of life satisfaction (42) on negative life events in adolescents. On the other hand, it also confirms that the influence of life satisfaction on self-injury can be affected by the risk factor of negative life events. Between the T2 and T3 time points, the relationship between negative life events and life satisfaction is not significant. It is possible that new life events have occurred, which may have had a fresh impact on life satisfaction, thereby masking the potential influence of negative events at T2 on life satisfaction at T3.

Finally, a cross-time mediation effect was identified: life satisfaction at T1 negatively predicted the occurrence of negative life events at T2, which in turn influenced non-suicidal self-injury at T3. This finding underscores the importance of life satisfaction as a protective factor against the development of NSSI. By promoting life satisfaction, it may be possible to reduce the likelihood of experiencing negative life events, which in turn can decrease the risk of engaging in NSSI.

### 4.4 Prevention implications

Firstly, recognizing the lagged effect of negative life events on NSSI underscores the importance of early identification and intervention. Schools and healthcare providers should be vigilant in recognizing signs of distress among adolescents and providing timely support, including access to mental health services, when needed.

Secondly, given that negative life events emerge as a significant risk factor for NSSI in adolescence, interventions aimed at preventing or mitigating these events could be effective in reducing NSSI behaviors. This includes providing adolescents with coping skills to manage stress and adversity, as well as fostering supportive environments at home, school, and within the community.

Finally, enhancing life satisfaction among adolescents is an important preventive strategy. This can be achieved through various interventions such as promoting positive coping strategies, increasing social support networks, and fostering resilience. School-based programs that incorporate mindfulness, stress management, and life skills training can be particularly effective in enhancing adolescents’ overall well-being and satisfaction with life.

### 4.5 Limitation

Although this study finds that negative life events and life satisfaction affect adolescents’ self-injury over time, there are still some limitations in this study: Firstly, this study investigated 268 junior high school students, only 208 students participated in three follow-up studies, the number of samples is small, future research should increase the sample to further improve the reliability of the study. Secondly, only Chinese were investigated in this study. Previous studies have found that the incidence and degree of adolescents’ self-injury are different in different cultures of the East and the West, so future studies should further consider the impact of cultural differences. Thirdly, this study adopts adolescents’ self-report method to investigate, there may be some reporting bias (50). To mitigate this issue, future research could consider incorporating multiple sources of information. Fourth, this study is the limited scope of psychiatric assessments, excluding conditions like depression, anxiety, personality issues, ADHD, ASD, or other psychiatric conditions, which may have influenced the observed relationships between self-injury and other variables. Future research should incorporate a broader psychiatric evaluation to provide a more comprehensive understanding of the issue. Fifth, we used only a simplified three-question measure of self-injury, which may lead to under-reporting of NSSI behavior. Future studies could use validated and comprehensive measurement scales to more accurately assess NSSI behaviors and their correlates. Lastly, Only three follow-up sessions for one and a half years was conducted, and the impact of life events on adolescents is always happening. Future research can use shorter intervals, more times and longer tracking time to collect data to explore the development of adolescents.

## 5 Conclusion

The findings of this study are as follows: (a) Negative life events have a cross-temporal impact on adolescents’ NSSI. Even if negative life events have occurred for half a year, they may become a trigger for adolescents’ self-injuries. (b) Life satisfaction also has a cross-time effect on the occurrence of life events among adolescents. Teenagers with lower life satisfaction may be more likely to experience negative life events and be troubled by it. (c) Life satisfaction can affect the occurrence of adolescents’ NSSI by influencing the experience of negative life events.

## Data Availability

The datasets for this article are not publicly available due to concerns regarding participant/patient anonymity. Requests to access the datasets should be directed to the corresponding author linlin@tjnu.edu.cn.

## References

[1] Nock MK. Self-injury. Annu Rev Clin Psychol. (2010) 6:339–63. doi: 10.1146/annurev.clinpsy.121208.131258 20192787

[2] Tan AC Rehfuss MC Suarez EC Parks-Savage A. Nonsuicidal self-injury in an adolescent population in Singapore. Clin Child Psychol Psychiatry. (2014) 19:58–76. doi: 10.1177/1359104512467273 23209310

[3] Hornor G. Nonsuicidal self-injury. J Pediatr Health Care. (2016) 30:261–7. doi: 10.1016/j.pedhc.2015.06.012 27094986

[4] Swannell SV Martin GE Page A Hasking P St John NJ. Prevalence of nonsuicidal self-injury in nonclinical samples: Systematic review, meta-analysis and meta-regression. Suicide Life-Threatening Behav. (2014) 44:273–303. doi: 10.1111/sltb.2014.44.issue-3 24422986

[5] Denton EG Álvarez K. The global prevalence of nonsuicidal self-injury among adolescents. JAMA Network Open. (2024) 7:e2415406–e2415406. doi: 10.1001/jamanetworkopen.2024.15406 38874928

[6] Qu D Wen X Liu B Zhang X He Y Chen D. Non-suicidal self-injury in Chinese population: a scoping review of prevalence, method, risk factors and preventive interventions. Lancet Regional Health Western Pac. (2023) 37:100794. doi: 10.1016/j.lanwpc.2023.100794 PMC10485683 37693882

[7] Muehlenkamp JJ Brausch AM. Body image as a mediator of non-suicidal self-injury in adolescents. J Adolescence. (2012) 35:1–9. doi: 10.1016/j.adolescence.2011.06.010 21777971

[8] Tang J Yang W Ahmed NI Ma Y Liu H Wang J. Stressful life events as a predictor for nonsuicidal self-injury in Southern Chinese adolescence: a cross-sectional study. Medicine. (2016) 95:e2637. doi: 10.1097/MD.0000000000002637 26945351 PMC4782835

[9] Liu X Liu L Yang J Zhao G. Reliability and validity of the adolescents self-rating life events checklist. Chin J Clin Psychol. (1997) 5:34–6.

[10] McFeeters D Boyda D Siobhan O. Patterns of stressful life events: distinguishing suicide ideators from suicide attempters. J Affect Disord. (2015) 175:192–8. doi: 10.1016/j.jad.2014.12.034 25638792

[11] Wang Y Sareen J Afifi TO Bolton S-L Johnson EA Bolton JM. A population-based longitudinal study of recent stressful life events as risk factors for suicidal behavior in major depressive disorder. Arch Suicide Res. (2015) 19:202–17. doi: 10.1080/13811118.2014.957448 25559346

[12] Liu RT Cheek SM Nestor BA. Non-suicidal self-injury and life stress: A systematic meta-analysis and theoretical elaboration. Clin Psychol Rev. (2016) 47:1–14. doi: 10.1016/j.cpr.2016.05.005 27267345 PMC4938721

[13] Grant KE Compas BE Stuhlmacher AF Thurm AE McMahon SD Halpert JA. Stressors and child and adolescent psychopathology: moving from markers to mechanisms of risk. Psychol Bull. (2003) 129:447–66. doi: 10.1037/0033-2909.129.3.447 12784938

[14] Hasking P Andrews T Martin G. The role of exposure to self-injury among peers in predicting later self-injury. J Youth Adolescence. (2013) 42:1543–56. doi: 10.1007/s10964-013-9931-7 23435860

[15] Madge N Hawton K McMahon EM Corcoran P De Leo D De Wilde EJ. Psychological characteristics, stressful life events and deliberate self-harm: findings from the Child & Adolescent Self-harm in Europe (CASE) Study. Eur Child Adolesc Psychiatry. (2011) 20:499–09. doi: 10.1007/s00787-011-0210-4 21847620

[16] Tanner A Hasking P Martin G. Co-occurring non-suicidal self-injury and firesetting among at-risk adolescents: experiences of negative life events, mental health problems, substance use, and suicidality. Arch Suicide Res. (2016) 20:233–49. doi: 10.1080/13811118.2015.1008162 26214360

[17] Nock MK. Why do people hurt themselves? New insights into the nature and functions of self-Injury. Current Directions in Psychological Science (2009). (2), 78–83. doi: 10.1111/j.1467-8721.2009.01613.x PMC2744421 20161092

[18] Guerry JD Prinstein MJ. Longitudinal prediction of adolescent nonsuicidal self-injury: Examination of a cognitive vulnerability-stress model. J Clin Child Adolesc Psychol. (2009) 39:77–89. doi: 10.1080/15374410903401195 PMC4626882 20390800

[19] Hankin BL Abela JR. Nonsuicidal self-injury in adolescence: Prospective rates and risk factors in a 2 ½ year longitudinal study. Psychiatry Res. (2011) 186:65–70. doi: 10.1016/j.psychres.2010.07.056 20807667 PMC3008214

[20] Liu RT Frazier EA Cataldo AM Simon VA Spirito A Prinstein MJ. Negative life events and non-suicidal self-injury in an adolescent inpatient sample. Arch Suicide Res. (2014) 18:251–8. doi: 10.1080/13811118.2013.824835 PMC4108499 24712970

[21] Morgan S. Poor psychological health and stressful-life events are more common in adolescents with self-harm thoughts or episodes. Evidence-Based Ment Health. (2012) 15:35–5. doi: 10.1136/ebmental-2011-100491 22398150

[22] Burke TA Hamilton JL Abramson LY Alloy LB. Non-suicidal self-injury prospectively predicts interpersonal stressful life events and depressive symptoms among adolescent girls. Psychiatry Res. (2015) 228:416–24. doi: 10.1016/j.psychres.2015.06.021 PMC4540325 26165966

[23] Diener E Emmons RA Larsen RJ Griffin S. The satisfaction with life scale. J Pers Assess. (1985) 49:71–5. doi: 10.1207/s15327752jpa4901_13 16367493

[24] Horley J. Life satisfaction, happiness, and morale: two problems with the use of subjective weil-being indicators. Gerontol. (1984) 24:124–7. doi: 10.1093/geront/24.2.124 6724315

[25] Headey B Kelley J Wearing A. Dimensions of mental health: Life satisfaction, positive affect, anxiety and depression. Soc Indic Res. (1993) 29:63–82. doi: 10.1007/BF01136197

[26] Borzumato-Gainey C Kennedy A McCabe B Degges-White S. Life satisfaction, self-esteem, and subjective age in women across the life span. Adultspan J. (2009) 8:29–42. doi: 10.1002/j.2161-0029.2009.tb00055.x

[27] Diener E Diener M. Cross-cultural correlates of life satisfaction and self-esteem. J Pers Soc Psychol. (1995) 68:653–63. doi: 10.1037/0022-3514.68.4.653 7738768

[28] Mahmoud JSR Staten RT Hall LA Lennie TA. The relationship among young adult college students’ depression, anxiety, stress, demographics, life satisfaction, and coping styles. Issues Ment Health Nurs. (2012) 33:149–56. doi: 10.3109/01612840.2011.632708 22364426

[29] St. John PD Mackenzie C Menec V. Does life satisfaction predict five-year mortality in community-living older adults? Aging Ment Health. (2015) 19:363–70. doi: 10.1080/13607863.2014.938602 25048721

[30] Wu L. Group integrative reminiscence therapy on self-esteem, life satisfaction and depressive symptoms in institutionalised older veterans. J Clin Nurs. (2011) 20:2195–203. doi: 10.1111/j.1365-2702.2011.03699.x 21631615

[31] Adrian M Zeman J Erdley C Lisa L Sim L. Emotional dysregulation and interpersonal difficulties as risk factors for nonsuicidal self-injury in adolescent girls. J Abnormal Child Psychol. (2011) 39:389–400. doi: 10.1007/s10802-010-9465-3 20953828

[32] Kress VE Newgent RA Whitlock J Mease L. Spirituality/religiosity, life satisfaction, and life meaning as protective factors for nonsuicidal self-injury in college students. J Coll Couns. (2015) 18:160–74. doi: 10.1002/jocc.2015.18.issue-2

[33] Rönkä AR Taanila A Koiranen M Sunnari V Rautio A. Associations of deliberate self-harm with loneliness, self-rated health and life satisfaction in adolescence: Northern Finland Birth Cohort 1986 Study. Int J Circumpolar Health. (2013) 72:21085. doi: 10.3402/ijch.v72i0.21085 PMC3753134 23984286

[34] Rotolone C Martin G. Giving up self-injury: A comparison of everyday social and personal resources in past versus current self-injurers. Arch Suicide Res. (2012) 16:147–58. doi: 10.1080/13811118.2012.667333 22551045

[35] Bishop AJ Martin P Randall GK MacDonald M Poon L. Exploring life satisfaction in exceptional old age: the mediating role of positive and negative affect. Clin Gerontol. (2012) 35:105–25. doi: 10.1080/07317115.2011.646389

[36] Lazić M Gavrilov-Jerković V Jovanović V. The moderating role of trait affect in the relationship between negative life events and life satisfaction. J Happiness Stud. (2018) s1:1–17. doi: 10.1007/s10902-018-0050-8

[37] Luhmann M Hofmann W Eid M Lucas RE. Subjective well-being and adaptation to life events: a meta-analysis. J Pers Soc Psychol. (2012) 102:592. doi: 10.1037/a0025948 22059843 PMC3289759

[38] Marum G Clench-Aas J Nes RB Raanaas RK. The relationship between negative life events, psychological distress and life satisfaction: a population-based study. Qual Life Res. (2014) 23:601–11. doi: 10.1007/s11136-013-0512-8 24026629

[39] Tian X. Negative life events and life satisfaction in university students: Belief in a just world as a mediator and moderator. J Health Psychol Pressed Online. (2016) 24(4):526–34. doi: 10.1177/1359105316678054 27852884

[40] Lin H-W Tung H-J. Using changes in life satisfaction and health to predict the survival status among older men and women in Taiwan. J Women Aging. (2013) 25:227–41. doi: 10.1080/08952841.2013.791600 23767838

[41] Siahpush M Spittal M Singh GK. Happiness and life satisfaction prospectively predict self-rated health, physical health, and the presence of limiting, long-term health conditions. Am J Health Promotion. (2008) 23:18–26. doi: 10.4278/ajhp.061023137 18785370

[42] Luhmann M Lucas RE Eid M Diener E. The prospective effect of life satisfaction on life events. Soc psychol Pers Sci. (2013) 4:39–45. doi: 10.1177/1948550612440105

[43] Wootton RE Davis OS Mottershaw AL Wang RAH Haworth CM. Genetic and environmental correlations between subjective wellbeing and experience of life events in adolescence. Eur Child Adolesc Psychiatry. (2017) 26:1119–27. doi: 10.1007/s00787-017-0997-8 PMC5591350 28508957

[44] Gorsuch RL. Exploratory factor analysis: Its role in item analysis. J Pers Assess. (1997) 68(3):532–60. doi: 10.1207/s15327752jpa6803_5 16372866

[45] Xiong C Xu Y. Reliability and validity of the Satisfaction with Life Scale for Chinese people. China J Health Psychol. (2009) 17:948–9. doi: 10.13342/j.cnki.cjhp.2009.08.026

[46] Rubin DB. Multiple imputation for nonresponse in surveys Vol. 81. New York: John Wiley & Sons (2004).

[47] Carlin J. Multiple imputation: a perspective and historical overview. In: Handbook of Missing Data. (London, United Kingdom: Chapman and Hall) (2015).

[48] Muthén LK Muthén BO. MPlus: statistical analysis with latent variables–User’s guide. (Los Angeles: Muthén & Muthén). (2012).

[49] Brown T. Confirmatory factor analysis for applied research. New York: The Guildford Press (2015).

[50] Podsakoff PM MacKenzie SB Lee J-Y Podsakoff NP. Common method biases in behavioral research: A critical review of the literature and recommended remedies. J Appl Psychol. (2003) 88:879–903. doi: 10.1037/0021-9010.88.5.879 14516251

[51] Barrocas AL Hankin BL Young JF Abela JR. Rates of nonsuicidal self-injury in youth: age, sex, and behavioral methods in a community sample. Pediatrics. (2012) 130:39–45. doi: 10.1542/peds.2011-2094 22689875 PMC3382916

[52] Whitlock J. 140. Non-suicidal self-injury in adolescents and young adults: general trends and gender differences. J Adolesc Health. (2011) 48:S90.

[53] Whitlock J Muehlenkamp J Eckenrode J Purington A Abrams GB Barreira P. Nonsuicidal self-injury as a gateway to suicide in young adults. J Adolesc Health. (2013) 52:486–92. doi: 10.1016/j.jadohealth.2012.09.010 23298982

[54] Xavier A Cunha M Pinto-Gouveia J. Daily peer hassles and non-suicidal self-injury in adolescence: gender differences in avoidance-focused emotion regulation processes. J Child Family Stud. (2018) 27:59–68. doi: 10.1007/s10826-017-0871-9

[55] Yue X Hiranandani NA Jiang F Hou Z Chen X. Unpacking the gender differences on mental health: the effects of optimism and gratitude. psychol Rep. (2017) 120:639–49. doi: 10.1177/0033294117701136 28558535

[56] Chapman AL Gratz KL Brown MZ. Solving the puzzle of deliberate self-harm: The experiential avoidance model. Behav Res Ther. (2006) 44:371–94. doi: 10.1016/j.brat.2005.03.005 16446150

[57] Heller D Watson D Ilies R. The role of person versus situation in life satisfaction: a critical examination. psychol Bull. (2004) 130:574. doi: 10.1037/0033-2909.130.4.574 15250814

[58] Bai Y Ben C Xu W Wu Y Liu Y. Reduce negative life events to Increase satisfaction: A daily diary study on the relationship between negative life events and life satisfaction. Soc Sci Med. (2024) 357:117191. doi: 10.1016/j.socscimed.2024.117191 39116698

